# Clinical analysis and prognostic factors in children with neuroblastoma-associated opsoclonus-myoclonus syndrome: a multicenter retrospective study

**DOI:** 10.1186/s12885-026-16198-6

**Published:** 2026-06-11

**Authors:** Chenjun Zheng, Chengliang Wan, Jinhu Wang, Yurui Wu, Weisong Cai, Tao Li, Qun Gao, Jianyao Wang, Shouhua Zhang, Xiuli Yuan, Bing Zhang, Bo Hai

**Affiliations:** 1 General Surgery Department, Kunming Children’s Hospital, No. 288, Qianxing Road, Xishan District, Kunming, Yunnan Province China; 2 Department of Surgical Oncology, Children’s Hospital, Zhejiang University of Medicine, No. 57, Zhugan Lane, Xiacheng District, Hangzhou, Zhejiang China; 3 Department of Thoracic and Oncologic Surgery, Capital Medical University, No. 2, Yabao Road, Chaoyang District, Beijing, China; 4 Department of Oncology, Shengjing Hospital of China Medical University, No. 36, Sanhao Street, Heping District, Shenyang, Liaoning China; 5 Department of Oncology, Children’s Hospital of Nanjing Medical University, No. 72, Guangzhou Road, Gulou District, Nanjing, Jiangsu China; 6 Anhui provincial Children’s Hospital, No. 39, Wangjiang East Road, Baohe District, Hefei, Anhui China; 7 Shenzhen Children’s Hospital, No. 7019, Yitian Road, Futian District, Shenzhen, Guangdong China; 8 Department of General Surgery, Jiangxi provincial Children’s Hospital, No. 122, Yangming Road, Donghu District, Nanchang, Jiangxi China; 9 Department of General Surgery, Fujian Children’s Hospital, No. 966, Hengyu Road, Jin’an District, Fuzhou, Fujian China

**Keywords:** Opsoclonus-Myoclonus Syndrome, Neuroblastoma, Paraneoplastic Syndromes, Prognosis, Immunotherapy, Pediatrics

## Abstract

**Background:**

The long-term neurological outcomes for children diagnosed with neuroblastoma associated with Opsoclonus Myoclonus Syndrome (NB-OMS) are not well characterized, primarily due to the infrequency of the condition. This multicenter study sought to evaluate the rate of neurological recovery and the long-term neurodevelopmental sequelae within this patient population.

**Methods:**

A retrospective study at nine Chinese medical centers (January 2015 - January 2025) involved 39 children with NB-OMS. Researchers gathered data on demographics, clinical presentation, tumor traits, treatment, and outcomes. Non-parametric tests and multivariate logistic regression identified prognostic factors.

**Results:**

In this study, a cohort of 39 patients was examined, with a median age at onset of 22 months. The retroperitoneum was identified as the most prevalent tumor location, accounting for 79.5% of cases. The majority of tumors were classified within the low-risk and intermediate risk, as determined by the International Neuroblastoma Risk Group Staging System, comprising 87.2% of the sample. Follow-up was conducted for 37 patients, all of whom exhibited symptom improvement, resulting in a 100% survival rate. Nonetheless, neurological sequelae were detected in 16 children (43.2%). A higher OMS severity score at onset demonstrated a strong trend towards significance (OR = 1.42, 95% CI: 1.00-2.01, *P =* 0.051). Multivariate analysis revealed that delayed initiation of treatment, defined as greater than 30 days, was a significant independent risk factor for the development of sequelae (Odds Ratio [OR] = 22.98, 95% Confidence Interval [CI]: 2.67-197.63, *P =* 0.004). Additionally, relapse of opsoclonus-myoclonus syndrome emerged as a significant independent risk factor (OR = 13.74, 95% CI: 1.37-137.34, *P =* 0.026). Furthermore, the use of combination immunotherapy, consisting of corticosteroids and intravenous immunoglobulin (IVIG), was associated with a significantly reduced median time to symptom resolution compared to non-combination therapy (5 months versus 8.5 months, *P =* 0.001).

**Conclusion:**

OMS is a chronic immune disorder often misdiagnosed, leading to treatment delays. Prompt first-line immunotherapy can quickly alleviate OMS symptoms. Early treatment and reducing relapses are crucial for better long-term outcomes. Future multicenter studies are needed to confirm the effectiveness of new immunotherapies.

## Introduction

Opsoclonus-myoclonus syndrome (OMS) (also known as dancing eye syndrome, Opsoclonus-myoclonus-ataxia syndrome, or Kinsbourne syndrome) is a rare but severe neurological disorder. It mainly presents with persistent, irregular, multidirectional saccadic eye movements (opsoclonus), myoclonus of the trunk and limbs, as well as ataxia and encephalopathic symptoms (such as irritability, insomnia). The annual incidence of this disease in children is extremely low, approximately 0.18–0.40 cases per million persons [1, 2]. The median age at onset is about 18–24 months. Approximately 50% of childhood OMS cases are associated with neuroblastoma (NB), making it an important paraneoplastic syndrome.

Non-paraneoplastic OMS in children can be closely associated with related antibodies such as anti-Hu, anti-Yo, and anti-SOX1. Paraneoplastic OMS in adults (e.g., breast cancer-related) is also frequently associated with anti-Ri antibodies. The specific autoantibody for childhood neuroblastoma-associated OMS (NB-OMS) has not yet been identified, which poses challenges for its diagnosis and treatment. Although NB-OMS usually has low-risk, localized, well-differentiated biological characteristics, and the tumor itself has an excellent prognosis, neurological sequelae are extremely common and severe, such as cognitive impairment, motor dysfunctions, language/speech abnormalities, which seriously affect the long-term quality of life of affected children [3].

Clinicians managing NB-OMS must address two main concerns: the primary tumor and related neurological impairment. Current treatment options include surgical resection combined with immunomodulatory agents such as corticosteroids, intravenous immunoglobulin (IVIG), or cyclophosphamide/rituximab. However, due to the low incidence of the disease, existing evidence on diagnosis and treatment is mostly based on small case series or reports; the optimal therapeutic regimen for NB-OMS has not yet been established. Therefore, to thoroughly examine the clinical features, treatment approaches, and long-term prognosis of children with NB-OMS, this study aims to summarize the diagnosis and treatment experiences from the past decade through a multicenter retrospective analysis, in order to provide an evidence-based foundation for clinical practice of this disease.

## Objects and methods

The study subjects were children diagnosed with neuroblastoma who were admitted to nine pediatric medical centers in China from January 2015 to January 2025. The participating centers were: Children’s Hospital of Zhejiang University School of Medicine, Jiangxi Provincial Children’s Hospital, Kunming Children’s Hospital, Anhui Medical University Children’s Medical Center, Shenzhen Children’s Hospital, Hunan Children’s Hospital, the Capital Center for Children’s Health at Capital Medical University, Shengjing Hospital of China Medical University, and Children’s Hospital of Nanjing Medical University. The criteria for inclusion were age at diagnosis under 18 years. The diagnostic criteria for OMS (included three of the following four manifestations): (a) Opsoclonus, (b) Myoclonus or ataxia, (c) Behavioral or sleep disturbance, (d) Neuroblastoma. Collected data included age, gender, age at onset, history of infection or vaccination, clinical symptoms, OMS severity at onset, antibody screening, tumor size and location, laboratory results, lymph node invasion, metastasis, pathological type, genetic test results, treatment, and prognosis follow-up. Clinical data were obtained from outpatient and inpatient records. The prognosis-related data were obtained through telephone interviews and outpatient follow-up. Children with less than 6 months of follow-up, as well as those with incomplete clinical data or misdiagnosed OMS, were excluded. Finally, 39 children with neuroblastoma who met the OMS diagnostic criteria were included in this analysis.

In addition for children aged 18 months or less: able to hold head consistently erect when trunk vertical? able to reach and grasp object with each hand? able to roll back to front and front to back? able to finger-feed self?

The OMS severity was assessed using the Mitchell and Pike OMS Rating Scale [4] (Table 1). Subjects were staged according to the International Neuroblastoma Risk Group Staging System(INRGss) [5, 6], classified into low risk(LR), intermediate risk(IR), and high risk(HR). Immunotherapy was administered in accordance with the dosing regimens specified in the expert consensus; the completion of the specified dosage was defined as one treatment course [7]. When a study object had an OMS score of zero for more than 3 months after treatment and had no sequelae such as motor, language, or intellectual decline, the child was considered to have no sequelae; otherwise, they were judged to have sequelae. Relapse of OMS was defined as the reappearance of symptoms lasting ≥ 48 h after complete remission for 1 month [8].

**Table 1:** OMS severity score by Drs. Wendy G. Mitchell and Michael G. Pike

Stance
0 standing and sitting balance normal for age
1 mildly unstable standing for age, slightly wide-based
2 unable to stand without support but can sit without support
3 unable to sit without using hands to prop or other support
Gait
0 walking normal for age
1 mildly wide-based gait for age but able to walk indoors and outdoors independently
2 walks only or predominantly with support from person or equipment
3 unable to walk even with support from person or equipment
Arm and hand function
0 normal for age
1 mild infrequent tremor or jerkiness without functional impairment
2 fine motor function (e.g., pincer grip of small object, pencil use) persistently impaired for age but less precise manipulative tasks (e.g., playing with larger toys feeding, dressing) normal or almost normal.
3 major difficulty with all age-appropriate manipulative tasks
Opsoclonus
0 none
1 rare or only when elicited by change in fixation
2 frequent, interfering frequently with fixation and/or tracking
3 persistent, interfering continuously with fixation and tracking
Mood/behaviour
0 normal
1 mild increase in irritability but consolable and/or mild sleep disturbance but easily settled
2 irritability and sleep disturbance, interfering substantially with child and family life
3 persistent severe distress

### Statistical methods

All data were statistically analyzed with SPSS 26.0 software. Measurement data were expressed as median and quartiles [Q1, Q3], and the Mann-Whitney U test was used to compare differences between two groups; enumeration data were expressed as number of cases (%), and the χ^2^ test or Fisher’s exact test was used to perform intergroup comparisons. In all analysis, a two-sided *P* < 0.05 was considered.

statistically significant, whereas *P* < 0.1 was considered as the criterion for multi-variate logistic regression analysis.

This multicenter retrospective study was conducted in accordance with the principles of the Declaration of Helsinki. Ethical approval was obtained from the Ethics Committee of Kunming Children’s Hospital (Approval number: 2025-03-371-K01). Given the retrospective design and the use of anonymized data, the requirement for informed consent was waived by all participating institutions.

## Results

### Demographics and clinical information

Over the past decade, 39 children who met the diagnostic criteria were enrolled, comprising 19 males and 20 females. The median age at onset was 22 months (range: 4–50 months), with a median interval from symptom onset to hospital presentation of 14 days. Except for one 4-month-old infant who was hospitalized for the primary tumor and developed OMS symptoms such as opsoclonus and irritability during adjuvant chemotherapy, all other children were admitted for the evaluation of OMS-related symptoms, with tumors subsequently detected on imaging studies.

Myoclonus was present in all patients (100%), followed by ataxia (76.9%). The median pre-treatment OMS severity score was 6. Laboratory studies showed that neuron-specific enolase (NSE) elevation was the most common abnormality (72.2%). Other findings were infrequent, including isolated cases of CSF oligoclonal bands(1/22) and anti-Hu antibodies(1/22), with low rates of abnormality in CSF analysis, Lactate dehydrogenase(LDH), ferritin(SF), and urinary vanillylmandelic acid(VMA) (see Table 2).

**Table 2:** Demographic and Tumor Characteristics of NB-OMS Children (*n* = 39)

Demographic Characteristics			Overall	Tumor Characteristics	Overall
Age(mo), median[IQR]		22 [17–28]		**Tumor size(cm)**	3.4(0.6–7.8)
Male, n (%)			19(48.7)	**INRGss**,** n(%)**	
Onset to visit time (days)			14 [5–16]	L1	23(58.9)
3 mo before onset, n(%)				L2	13(33.3)
History of vaccination			4(10.3)	M	2(5.2)
Fever			3(7.7)	MS	1(2.6)
Clinical manifestations, n(%)				**Histology(INPC)**	
Myoclonus			39(100.0)	NB	24(61.5)
Opsoclonus			18(46.2)	GNBn	11(28.2)
Ataxia	30(76.9)			GNBi	4(10.3)
Behavioral/sleep disturbance			16(41.0)	**Lymph Node Positive**,** n(%)**	12(33.0)
OMS severity score			6,(3, 13)	**MYCN Positive**,** n(%)**	2(6.9)
Elevated LDH, n(%)			3/36(8.3)	**Distant Metastasis**,** n(%)**	3(7.7)
Elevated SF, n(%)			1/25(4.0)	**Risk Stratification**,** n(%)**	
Elevated NSE, n(%)			26/36(72.2)	LR	21(53.9)
Elevated VMA, n(%)			2/12(16.7)	IR	13(33.3)
Tumor Location, n(%)				HR	5(12.8)
adrenal area			18(46.2)		
Retroperitoneal area*			13(33.3)		
Posterior mediastinum			8(20.5)		

*INPC* International Neuroblastoma Pathology Classification, *INRGss* International Neuroblastoma Risk Group

*Retroperitoneal area excluding the adrenal area

The tumor was most frequently located in the retroperitoneum (79.5%), and one originated from the sacrococcygeal region. Neuroblastoma was the predominant pathological type (61.5%), with subtypes including: poorly differentiated type 13 cases, differentiated type 11 cases. Distant metastasis rate was 7.7%, involving sites such as liver, cervical lymph nodes, bone marrow, and brain. MYCN gene amplification was observed in 6.9% of tested cases. According to the INRGss, most cases were classified as L1 stage (58.9%) and low-risk (53.9%). Of the 15 patients tested for DNA ploidy, all exhibited a hyperdiploid genotype. Subsequent molecular analysis revealed an EGFR mutation in one case and two acquired copy number variations (CNVs) in another. Detailed demographic and tumor characteristics are summarized in Table 2.

### Treatment and prognosis

The median time from onset to treatment for the 39 children was 28 days. One child had spontaneous tumor regression during treatment. Due to the diffuse hepatic metastasis, one could not undergo surgery. The remaining 37 children all underwent surgical treatment. ROC curve analysis of the time from onset to treatment yielded an AUC value of 0.734, with 28.5 days as the optimal cutoff value. Therefore, within 30 days was defined as the early treatment group and more than 30 days as the late treatment group. Among them, 31 cases underwent primary surgery. Residual tumor was identified in 2 children during chemotherapy, both of whom subsequently underwent a second surgery 4 months later. 28 children received chemotherapy. Chemotherapy regimens were based on the risk group according to the expert consensus on diagnosis and treatment of childhood neuroblastoma [9, 10]. The low-risk group received 2–4 courses of chemotherapy, the intermediate-risk group received 4–6 courses of CBVP + CADO regimen chemotherapy. Three months post-surgery, genetic testing of one pediatric patient revealed a positive MYCN status, prompting an escalation of the chemotherapy regimen to a high-risk protocol. This protocol consisted of six courses of chemotherapy, each administered in conjunction with corticosteroids and IVIG, for a total of eight cycles of such adjunctive therapy throughout the treatment course. Despite the family’s decision to decline additional adjuvant therapies, such as radiotherapy, autologous stem cell transplantation, and GD2 immunotherapy, the patient achieved a favorable long-term outcome. At the 48-month follow-up, there was no evidence of tumor recurrence or neurological sequelae.

24 children received corticosteroids therapy (dexamethasone, methylprednisolone), with an average treatment duration of 4 months (1–15 months); 26 children received IVIG therapy, with an average treatment duration of 3.3 months (1–16 months); 4 children received rituximab therapy; there were no cases using adrenocorticotropic hormone (ACTH). The 4 cases using rituximab were all for symptoms with poor initial relief or relapse. We present the general characteristics, diagnosis, and treatment details of the high-risk group in the following table (Table 3).

**Table 3:** Characteristics and Treatment of High-Risk Patients

No	Sex	Age(mo)	OMS	INRGss	MYCN	Metastasis	Surgery	Chemo-therapy	Immuno-therapy	Sequela
1	F	15	12	L1	Y	N	Y	Y	C + I	N
2	F	21	3	L1	Y	N	Y	N	N	Y*
3	M	18	5	M	N	Y	Y	Y	C + I	N
4	F	14	3	L2	N	N	Y	Y	C + I	N
5	F	23	4	M	N	Y	Y	Y	N	-

*OMS* OMS severity score at onset, *C* corticosteroids, *F* female, *I* intravenous immunoglobulin, *M* male, *N* no, *Y* yes

* during intercurrent infections, she presented with mild, transient motor dysfunction, primarily manifesting as an increased tendency to fall

A total of 37 out of 39 children were followed up, with an average follow-up time of 46.6 months (range 7-125 months). No deaths occurred during the follow-up period for all patients, with a survival rate of 100% and an event-free survival rate of 94.6%. OMS symptoms significantly improved in all treated children. OMS symptoms recurred in 10 cases (27.0%); Symptoms recurred more than three times in three children. One child’s symptoms resolved after treatment with corticosteroids + IVIG, while the remaining two were treated with a combination of corticosteroids + IVIG + rituximab.

It was ultimately found that 16 children (43.2%) still had varying degrees of neurological sequelae such as motor, language, and intellectual impairments. Factors that might affect the prognosis of NB-OMS children were correlated with sequelae (Tables 4 and 5). Factors with *P <* 0.1 were included in a multivariate Logistic regression analysis, ultimately obtaining factors related to the prognosis of NB with OMS (Table 6).

**Table 4:** Correlation Analysis of Demographics, Treatment, and Sequelae in NB-OMS Children (*n* = 37)

Characteristic	Overall		With Sequelae	Without Sequelae	*P* Value	
Gender, n (%)					0.886	
Male	19(51.4)		8(50)	11(52.4)		
Female	18(48.6)		8(50)	10(47.6)		
Age at onset (mo)	21[17, 27]		22[18, 27]	21[17, 28]	0.421	
OMS severity score			5[4, 7]	7.5[6, 9]	0.032	
Opsoclonus					0.191	
Yes	18(48.6)		10(62.5)	8(38.1)		
No	19(51.4)		6(37.5)	13(61.9)		
Ataxia					0.248	
Yes	28(75.7)		14(87.5)	14(66.7)		
No	9(24.3)		2(12.5)	7(33.3)		
Time to treatment						0.001
Early treatment		21(56.8)		4(25.0)	17(81.0)	
Late treatment		16(43.2)		12(75.0)	4(19.0)	
Corticosteroid therapy						0.739
Yes		24(64.9)		11(68.8)	13(61.9)	
No		13(35.1)		5(31.3)	8(38.1)	
IVIG treatment						0.491
Yes		25(67.6)		12(75.0)	13(61.9)	
No		12(32.4)		4(25.0)	8(38.1)	
Chemotherapy						1.000
Yes		27(73)		12(75.0)	15(71.4)	
No		10(27)		4(25.0)	6(28.6)	
Relapse of OMS						0.009
Yes		10(27.0)		8(50)	2(9.5)	
No		27(73.0)		8(50)	19(90.5)	

**Table 5:** Correlation Analysis of Oncological Factors Affecting Sequelae in NB-OMS Children (*n* = 37)

Characteristic	Overall	With Sequelae	Without Sequelae	*P* Value	
Tumor location, n (%)				0.596	
Suprarenal gland	17(45.6)	6(37.5)	11(52.4)		
Retroperitoneum area	13(35.1)	6(37.5)	7(33.3)		
Posterior mediastinum	7(18.9)	4(25.0)	3(14.3)		
Tumor diameter (cm)	3.4 ± 1.4	3.2 ± 1.5	3.5 ± 1.4	0.605	
Histopathologic type, n (%)				0.877	
NB	23(62.2)	11(68.8)	12(57.2)		
GNBn	11(29.7)	4(25)	7(33.3)		
GNBi	3(8.1)	1(6.3)	2(9.5)		
INRGss, n (%)				0.739	
LR	21(56.8)	10(62.5)	11(52.4)		
IR + HR	16(43.2)	6(37.5)	10(47.6)		
Degree of Differentiation, n (%)					1.000
Poorly Differentiated		12(60%)	6(60%)	6(60%)	
Differentiated	8(40%)	4(40%)	4(40%)		

**Table 6:** Multivariate Logistic Regression Analysis of Factors Affecting Prognosis in NB-OMS Children (*n* = 37)

Factor	*P* Value	Odds Ratio (OR)	95% Confidence Interval (CI)	
			Lower Limit	Upper Limit
OMS severity score	0.051	1.417	0.999	2.009
Time to treatment	0.004	22.976	2.671	197.629
Relapse of OMS	0.026	13.736	1.374	137.34

To investigate the relationship between immunotherapy and the duration until initial complete resolution of OMS symptoms, patients were categorized based on their treatment regimen. Based on the observation that corticosteroids and IVIG were predominantly used in combination in this cohort, patients who received both therapies were classified into a “Combination Therapy” group, which was compared against a “Non- Combination Therapy” group (comprising patients who received either monotherapy or no immunotherapy). This categorization resulted in a combination therapy cohort (*n* = 23) and a non-combination therapy cohort (*n* = 14). The baseline characteristics of these two cohorts were subsequently compared, as presented in Table 7.

**Table 7:** Comparison of Baseline Characteristics Between Combination and Non-Combination Immunotherapy Groups

Group	*n*	Age (mo)	Male	OMS Score	Chemo-therapy	NB Type	Early Treatment	LR
Combination Therapy	23	20[15,27]	12(63.2)	7[5.5,9.5]	9(39.1)	17(73.9)	15(65.2)	12(52.2)
Non-Combination Therapy	14	22[19,28]	7(50.0)	4[3,6.5]	4(28.6)	10(71.4)	6(42.8)	9(64.2)
Statistical Value		12.546	0.016	77.5	0.087			162.5
*P* value		0.403	0.898	0.008	0.724	1.000	0.305	0.515

*NB* neuroblastoma, *LR* Low Risk

Data are presented as n (%) for categorical variables

The two groups were comparable at baseline regarding ataxia (*P* = 1.000), opsoclonus (*P* = 0.769), and tumor diameter (*P* = 0.785). The assumption of normality was violated for one dataset, justifying the use of the non-parametric Mann-Whitney U test. This analysis indicated a statistically significant difference in the median time to symptom resolution between the combination therapy group (5 months) and the non-combination therapy group (8.5 months) (U = 265, *P =* 0.001).

## Discussion

Approximately 1.8% to 3.0% of children with neuroblastoma are also diagnosed with OMS [11], a rare paraneoplastic neuroimmune disorder that presents unique challenges in clinical management. This study conducts a systematic analysis of 39 pediatric patients diagnosed with neuroblastoma-associated opsoclonus-myoclonus syndrome (NB-OMS) to identify critical factors influencing their long-term prognosis, thereby providing valuable insights for the clinical management of this complex condition. The epidemiological characteristics of this cohort align with those reported in large-scale international studies. The median age of onset was 22 months, with 35 cases (89.7%) occurring before the age of 3 years, and no cases observed in children older than 5 years. Although this median age is slightly later than the commonly reported 18 months, it remains consistent with the high incidence observed in infancy and early childhood [12]. In the present study, no significant association was found between age and sequelae (*P* = 0.421).

The diagnosis of NB-OMS lacks specific biomarkers and relies on clinical recognition. In our study, the NSE exhibited a positivity rate of 72.2%, contrasting with the relatively low positivity rates observed for SF, LDH, and VMA. Among the 22 cases that underwent CSF examination, oligoclonal bands were detected in only one case, and one antibody test returned positive for the anti-Hu antibody. One study suggests that the presence of anti-GluD2 antibodies, particularly in patients who remain persistently positive without a known tumor, should prompt comprehensive and ongoing investigations to rule out underlying neuroblastoma [13]. Conversely, a subsequent larger study did not detect specific anti-GluD2 antibodies in any of the 203 OMS patients and 172 controls [14]. These conflicting findings suggest that the role of anti-GluD2 antibodies in OMS requires further validation through standardized and reproducible experiments. In light of the absence of clinical or laboratory markers that facilitate early differentiation from non-tumor-associated OMS, some studies recommend against routine CSF examination [15]. Nevertheless, in patients presenting with atypical clinical manifestations, who are at risk of being misdiagnosed with conditions such as encephalitis, acute cerebellar ataxia, or epilepsy, thereby complicating clinical diagnosis and management, CSF examination can be instrumental. It aids in excluding related pathologies and in the detection of B lymphocytes within the CSF. An increase in B lymphocytes can inform the administration of pharmacological agents such as cyclophosphamide and rituximab, which have been shown to ameliorate OMS symptoms in certain patients [16, 17].

A study indicated that the average duration for tumor detection was 365 days in children with sequelae, compared to 57 days in those without [18]. Another study conducted in the United States involving 356 children with OMS reported that the interval from symptom onset to diagnosis ranged from approximately 2.1 to 7.5 months [12]. Contrastingly, our research identified a significantly shorter median time from symptom onset to treatment initiation, at 28 days. In our cohort, with one exception, all tumors were diagnosed through examinations initiated by hospitalization due to OMS symptoms, largely attributable to markedly enhanced disease recognition among clinicians. Considering that at least 50% of childhood OMS is associated with neuroblastoma, which may not manifest until six months or more following OMS onset, it is advisable to conduct repeated serum, urine, and imaging assessments 4–6 months after the initial diagnosis and treatment. This practice aims to mitigate the risk of false negatives and prevent delays in surgical intervention [19].

Regarding tumor biological characteristics, one study [15] found that 93% of neuroblastomas in NB-OMS were early stage (L1 or L2). In this study, tumors were mainly distributed in the retroperitoneum (76.9%), INRGss was predominantly L1/L2 (92.2%), risk stratification concentrated in the low-risk and intermediate risk (87.2%), and MYCN amplification positivity was low (6.9%), once again confirming the strong correlation between NB-OMS and low-risk neuroblastoma. The systematic review and meta-analysis conducted by Xie et al. indicated that patients with mediastinal neuroblastoma-associated OMS exhibit more favorable clinical characteristics, necessitate less prolonged immunotherapy, and have a significantly reduced risk of persistent neurological symptoms compared to patients with non-mediastinal tumors [20]. However, our study does not corroborate these findings, as it shows no significant difference in the prevalence of neurological sequelae (4/7 vs. 12/30, *P* = 0.437) or in the time required for symptom resolution (*P* = 0.776) between the two groups. In this study, 37 children were followed up (the longest exceeding 10 years), with no cases of tumor recurrence or death, further supporting the excellent prognosis of NB-OMS. The reason for this difference may be related to the immune system’s role in clearing the tumor [20]. Some studies [21] suggest that NB-OMS has a higher proportion of the SCA genomic type, but its oncological prognosis is excellent, possibly related to anti-tumor immune responses. Genomic type does not have prognostic value in such patients, and the specific reasons require further study. 5 patients were categorized into the high-risk group. Follow-up data indicated that three of these patients had a follow-up duration exceeding 20 months, with one case exhibiting mild motor dysfunction. This duration constituted the primary observation period for this cohort. Furthermore, the two MYCN-positive patients, monitored for 48 and 13 months respectively, showed no evidence of tumor recurrence or sequelae; an increased incidence of neurological sequelae was not observed in children with high-risk neuroblastoma. Anand et al. [22] also observed that children with low-stage (stage 1/2) tumors exhibited a higher incidence of neurological sequelae. This finding may be ascribed to either a potentially heightened autoimmune response elicited by low-stage tumors, which, in targeting the tumor, concurrently causes more pronounced damage to the nervous system, or to the more aggressive combination therapy possibly eliminating the pertinent antibodies. However, due to the limited sample size, larger-scale studies are necessary to validate these findings.

There remains considerable scope for discussion regarding treatment strategies for NB-OMS. Some studies have indicated that surgical resection alone is insufficient for curing OMS, whereas a combination of dexamethasone and cyclophosphamide constitutes an effective and feasible therapeutic strategy, Early and aggressive treatment may mitigate cognitive and motor sequelae [23, 24]. Despite no significant reduction in sequelae following initial radical resection in this study (*P* = 0.202), our clinical observations suggested improvement in OMS symptoms post-operation in some children. Notably, two patients with residual tumors developed sequelae even after combined therapy and a reoperation. However, the relationship between surgical timing and sequelae requires validation through larger prospective trials. While the literature notes that spontaneous tumor regression can occur in a subset of NB-OMS cases and may be associated with a favorable neurologic outcome [25], clinical experience suggests that such an ideal course is not guaranteed. In the cohort studied by Anand et al. [22], pathologic findings suggestive of possible spontaneous regression were identified. In this study, a single patient, aged 7 months, received a treatment regimen comprising methylprednisolone, IVIG, and rituximab, following which the tumor demonstrated spontaneous regression; this patient did not exhibit any intellectual or language developmental impairments, with only transient myoclonus observed, triggered by events such as infections. Similarly, three patients achieved symptom relief and did not develop sequelae following surgical intervention combined with chemotherapy, in the absence of immunotherapy. Further investigation into the mechanisms linking neuroblastoma and OMS is essential to elucidate the pathogenesis and enhance interventions aimed at improving intellectual outcomes. However, given the limited number of cases treated with surgery and chemotherapy, and considering that unresolved or recurrent symptoms can result in severe neurological sequelae with significant impacts on quality of life, this study advocates for early and aggressive treatment as the more appropriate strategy.

This study demonstrated that the incidence of sequelae in the early treatment group (≤ 30 days) was significantly lower compared to the late treatment group (4/21 vs. 12/16, *P =* 0.001). A study [4] retrospectively analyzing 14 children with NB-OMS suggested that diagnostic delay (> 2 months) was significantly associated with memory and motor impairment (*P =* 0.032, *P =* 0.024), emphasizing the necessity of early intervention. From the pathophysiological mechanism perspective, this time window reflects the damage process of uncontrolled neuroimmune inflammation to the central nervous system. The prevailing hypothesis regarding the pathogenesis of OMS suggests a molecular mimicry mechanism, wherein tumor-expressed neural-related antigens elicit an autoimmune response against cerebellar Purkinje cells and brainstem neurons [18]. If this inflammatory process is not promptly addressed, it may result in irreversible neuronal damage and hinder neural network remodeling. Consequently, any delay in treatment effectively prolongs the duration of the autoimmune attack on the nervous system. This finding indicates that delays in any diagnostic step may directly impact the child’s long-term neurological functional outcomes. Therefore, it is recommended that patients presenting with suspected OMS-related symptoms undergo comprehensive full-body imaging examinations at the earliest opportunity to facilitate prompt diagnosis and treatment. In cases where the medical institution lacks the necessary treatment capabilities, timely referral is advised to prevent missing the optimal treatment window. Study [21] underscores that early, aggressive combined immunotherapy (e.g., intravenous dexamethasone, IVIG, and rituximab) can substantially reduce recurrence rates and prevent permanent neurological sequelae.

In this study, no correlation was found between immunotherapy and sequelae. This may be attributable to non-standardized early treatment and premature drug discontinuation in some cases. Among the 10 recurrent cases, only 2 completed an immunotherapy course of 6–12 months. The remaining 8 cases discontinued treatment on their own within 3 months post-operation upon symptom improvement, which subsequently led to OMS recurrence. Thus, our findings confirm that OMS recurrence is a reliable indicator for the development of long-term sequelae. The phenomenon of recurrence implies that initial immunotherapy may not have fully eradicated autoreactive immune cell clones, or that the child may possess an intrinsic defect in immune regulation. Common triggers for recurrence include infections and the tapering of treatment medications [8]. Sheridan et al. [26] conducted a study involving 37 children, which demonstrated that the number of relapses (*P <* 0.001) and persistent OMS symptoms were significantly correlated with poorer long-term cognitive outcomes. Specifically, each additional relapse was associated with an average decrease of 2.4 points in the Full-Scale Intelligence Quotient. A study conducted in the US involving 14 patients indicated that the early administration of rituximab, in conjunction with IVIG and adrenocorticotropic hormone, could mitigate the risk of relapse [27]. Another investigation reported that administering rituximab earlier (by two cycles) to OMS patients experiencing relapse resulted in improvements in neurological symptoms [28]. In a study by Mitchell et al., where 14 children initially received treatment with IVIG and steroids, 10 were subsequently administered rituximab and 5 received cyclophosphamide; the group receiving intensified therapy exhibited significantly better outcomes [17] in adaptive behavior, IQ, and motor function compared to the historical control group (*P <* 0.001). It has also been reported [29] that combining steroids and IVIG with second-line therapy early in the disease course may improve prognosis. Rapid steroid tapers can lead to OMS relapse and should be avoided. One study indicates that using rituximab early in an immunomodulatory protocol may significantly shorten corticosteroids and IVIG therapy duration without affecting short-term clinical outcomes [30]. In our cohort, 4 patients were administered rituximab, with two initiating treatment following three relapses. While complete remission of OMS symptoms was achieved, one patient exhibited mild motor deficits, and two experienced moderate language and motor impairments. This outcome is likely attributable to the initially more severe disease presentation in the children receiving rituximab. Consequently, early initiation of rituximab upon relapse, or its incorporation into an initial treatment regimen, appears to be a more efficacious strategy for mitigating long-term sequelae in children with severe symptoms.

A study conducted in the UK, with a 53-year follow-up, identified that severe initial symptoms significantly increased the risk of a chronic disease trajectory (OR = 2.77, *P =* 0.002) and learning disabilities (OR = 2.03, *P =* 0.026). The study advocated for early, intensive immunotherapy, such as rituximab, to potentially enhance outcomes, particularly in refractory cases [31]. The effectiveness of combination therapy for NB-OMS is substantiated by Level I evidence from the ANBL00P3 trial. This study demonstrated a significantly enhanced neurological response rate with IVIG-based therapy, achieving a response rate of 80.8% compared to 40.7% in the control group (Odds Ratio = 6.1, 95% confidence interval: 1.5–25.9, *P* = 0.0029).In this study, the observed significantly shorter duration to achieve OMS remission with combination therapy (5 months compared to 8.5 months, *P* = 0.001) substantiates the enhanced therapeutic efficacy of the combined regimen, demonstrating a median reduction in time to remission by 3.5 months. Following combination therapy, the time to remission was significantly reduced in children with OMS, indicating that a first-line immunotherapy regimen alone can be effective even in severe OMS score cases. Treatment strategies can be tailored according to the child’s symptomatic response.

In this study, 16 children (43.2%) still had varying degrees of neurological sequelae such as motor, language, and intellectual impairments.

In this study, 40.5% of the cases (*n* = 15) demonstrated mild motor dysfunction, while 18.9% (*n* = 7) exhibited mild intellectual and speech dysfunction. Although more aggressive immunotherapy, such as the combination of IVIG, steroids, and chemotherapy, may result in short-term improvements in neurological function and quality of life—enabling some patients to achieve symptom remission and functional stability—long-term neurocognitive, behavioral, and adaptive deficits remain highly prevalent [4]. Brunklaus et al. [31], in a more extensive retrospective study, corroborated these findings, reporting motor impairments in 60% of patients, speech abnormalities in 66%, learning disabilities in 51%, and behavioral problems in 46%, with only approximately one-third achieving normal intellectual outcomes and complete symptom resolution. Furthermore, the severity of these sequelae is closely linked to factors such as age at onset, initial symptom severity, treatment response, and whether the disease follows a chronic relapsing course. Consequently, despite the short-term benefits afforded by advancements in treatment strategies, the long-term prognosis remains uncertain and necessitates further validation through larger, prospective, long-term follow-up studies.

### Study limitations

This study’s retrospective design renders it vulnerable to information and selection bias, and its small sample size further limits its statistical power. Variations in treatment protocols across the multiple centers involved may have contributed to differences in observed outcomes. The young age of some patients posed challenges for formal intelligence testing, and the absence of standardized, objective quantitative measures for assessing sequelae may have introduced bias. Cognitive outcomes in this study were based on subjective assessments.

## Conclusion

OMS is a chronic, immune-mediated disorder that is frequently misdiagnosed, resulting in delayed treatment. Early administration of first-line immunotherapy can expedite symptomatic remission. Early intervention and the minimization of OMS relapses are essential for enhancing long-term outcomes. Future prospective multicenter studies are necessary to validate the efficacy of novel immunotherapies.

## Data Availability

The datasets generated and/or analysed during the current study are not publicly available due to restrictions stipulated in the data governance policies of some participating institutions in this multi-center study. However, the de-identified data supporting the key findings are available from the corresponding author on reasonable request and with permission from the relevant institutions.

## Abbreviations

NB: neuroblastoma
OMS: Opsoclonus Myoclonus Syndrome
NB-OMS: neuroblastoma associated Opsoclonus Myoclonus Syndrome
IVIG: intravenous immunoglobulin
INRGss: International Neuroblastoma Risk Group Staging System
LDH: Lactate dehydrogenase
NSE: neuron-specific enolase
SF: ferritin
VMA: vanillylmandelic acid
LR: low risk
IR: intermediate risk
HR: high risk
CNVs: copy number variations
SCA: Segmental Chromosome Alterations
